# Supplementary material for: Interpersonal physiological and psychological synchrony predict the social transmission of nocebo hyperalgesia between individuals
Source: Commun Psychol. 2024 Apr 29;2:33. doi: 10.1038/s44271-024-00069-6 (PMC11332037; doi:10.1038/s44271-024-00069-6)
Supplement: Supplementary file 1 — Supplementary Information [file 44271_2024_69_MOESM1_ESM.pdf]

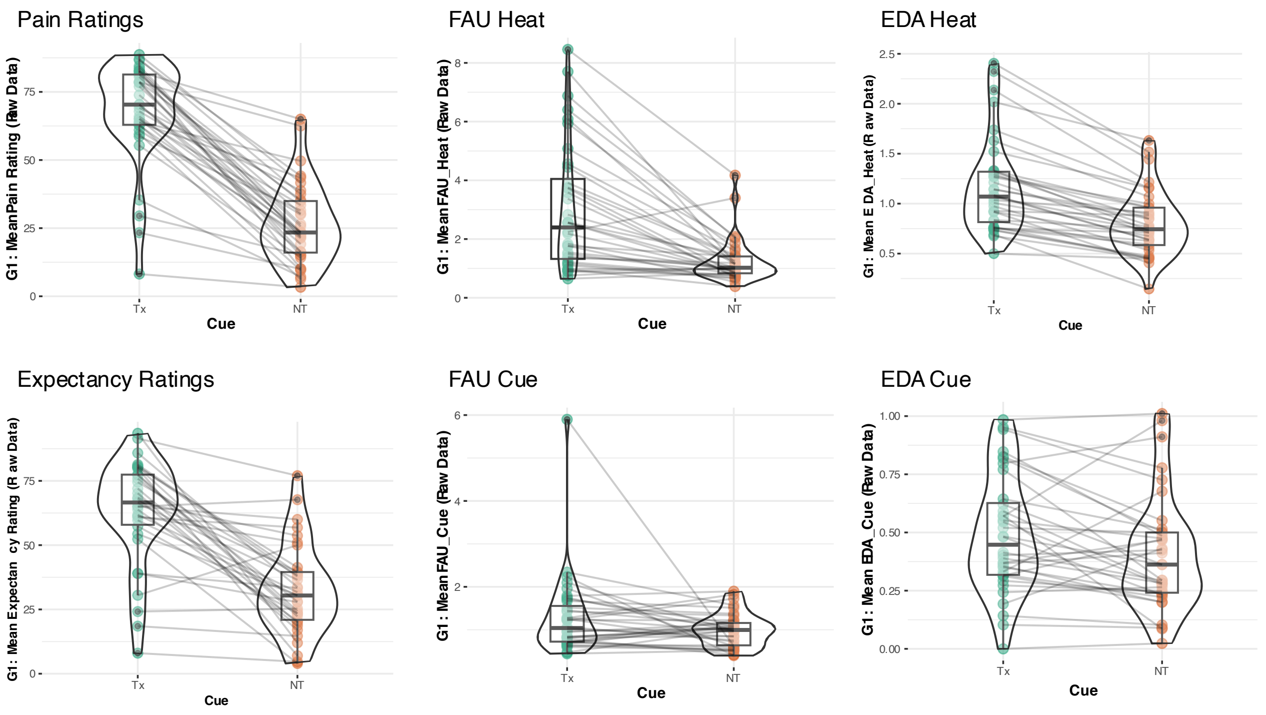

Supplementary Figure 1:
Violin plots depicting the raw Data for Primary and Secondary Outcomes (Generation 1 Demonstrators; *N*=36, except for 'Expectancy Ratings' where *N*=34 due to missing data). In these Figures Tx refers to 'Treatment' and NT to 'No Treatment'.

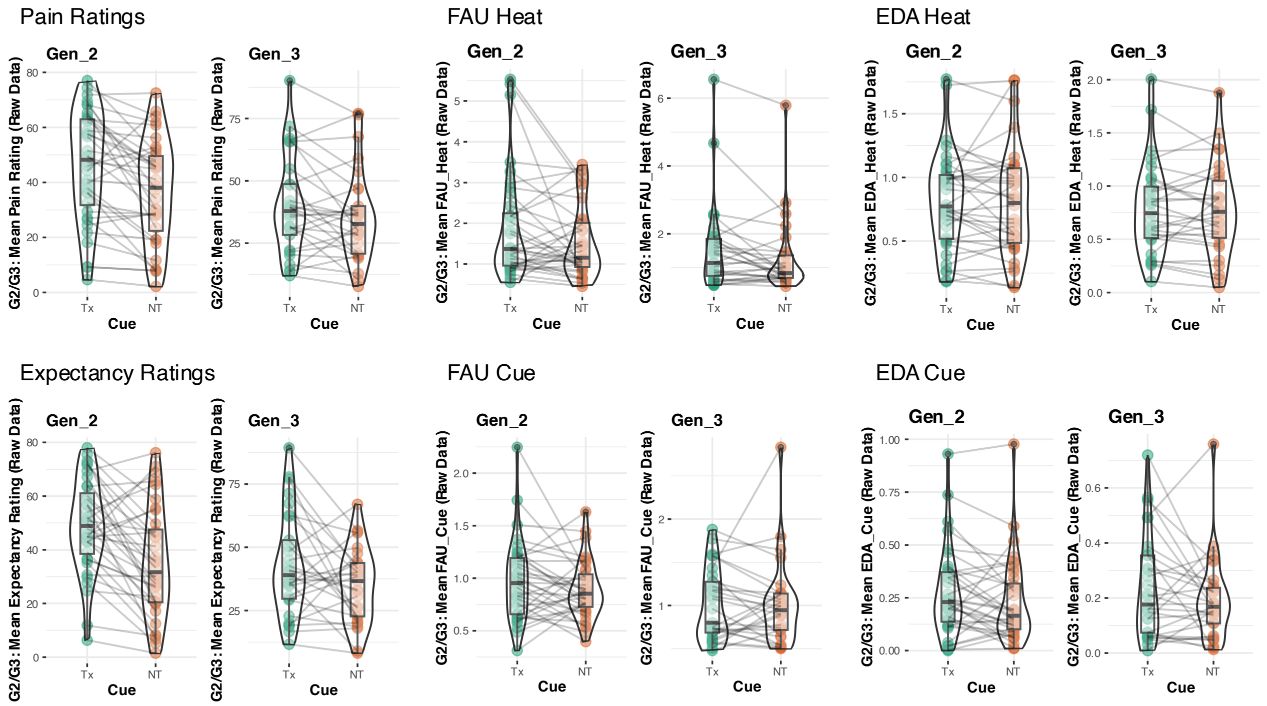

Supplementary Figure 2:
Raw Data for Primary and Secondary Outcomes (Generation 2 (*N*=36) and Generation 3 (*N*=29) Demonstrators). In these Figures Tx refers to 'Treatment' and NT to 'No Treatment'. Gen_2 refers to the Second Generation Participant and Gen_3 the Third Generation.

Supplementary Note 1: Manipulation Check (Generation 1 Demonstrators)

To ensure that the High and Low thermal stimuli were sufficient to generate differences in the G1 pain response to Tx and NT cues, all phasic outcomes (pain ratings, expectancy ratings, $FAU_{cue/heat}$ and $EDA_{cue/heat}$) were analysed via within-subjects ANCOVAs with Cue and Trial as factors and Gender-Match as the covariate (see: H_1).

As depicted in Supplementary Figure 1, a statistically significant main effect of Cue (Tx > NT) was found for all outcomes (all *ps*<.016), with the exception of FAU_{cue}, which neared statistical significance ($F(1, 34)=3.16$, $p=.085$, $\eta_p^2=.09$). For Pain and Expectancy ratings, there was an interaction between Cue and Trial (both *ps*<.001), with pain increasing to Tx and reducing to NT. Phasic responses to the thermal stimulus (FAU_{heat} and EDA_{heat}) demonstrated general habituation (main effect of Trial; both *ps*<.002) after the first two trials. No other main effects or interactions reached statistical significance (all *ps*>.05).

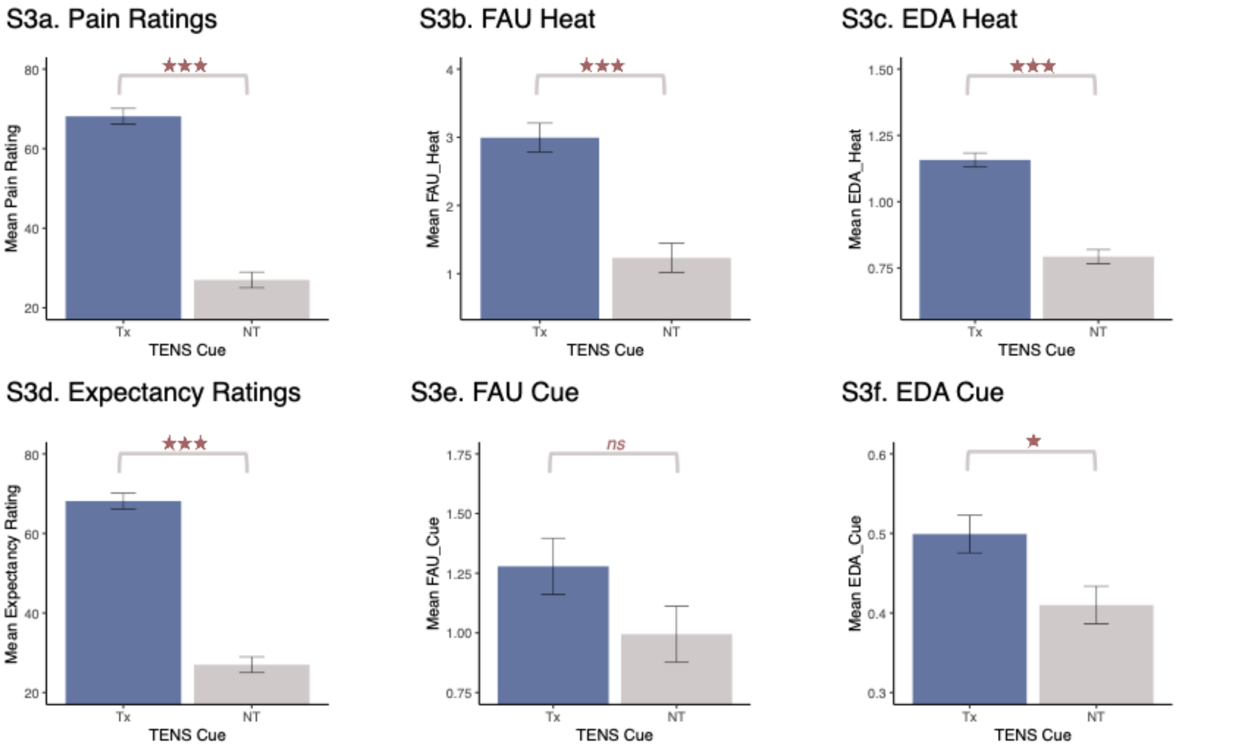

Supplementary Figure 3:
Mean Generation 1 Demonstrator responses to Treatment (paired with the High Temperature Destination; 54°C) and No Treatment (paired with the Low Temperature Destination; 45°C). Error bars represent +/- 1SED, adjusted for the within-subjects comparison (using the afex package in R). Significance levels are depicted as *** (p<.001); ** (p<.01), and * (p<.05). Tx refers to 'Treatment' and NT to 'No Treatment'. N=36, except for Expectancy ratings (N=34).

Supplementary Note 2: Analysis of Secondary Variables

Phasic measures of EDA_{cue}, EDA_{heat}, FAU_{cue} and FAU_{heat} were analysed using the same ANCOVA model as Pain Ratings (testing H_2-H_3). As missing data existed (e.g., due to removal of outliers; 60 trials total—average 0.3 trials per participant), to reduce listwise deletion sequential pairs of Trials (Tx and NT) were averaged together (resulting in a within-subject factor of Trial with three levels: 1 (average of trials 1-2); 2 (average of trials 3-4); 3 (average of trials 5-6)).

Anticipatory Outcomes: FAU Cue

There were no statistically significant main effects or interactions for FAU_{cue} (all *ps*>.05). This is arguably unsurprising given that the anticipatory pain-related facial expressions of G1 participants also did not significantly differ between Tx and NT Cues.

Anticipatory Outcomes: EDA Cue

An anticipatory autonomic response was observed to the Tx (M_{adj}=0.25, *SEM*=0.03) relative to NT (M_{adj}=0.21, *SEM*=0.02) Cue ($F(1, 62)=4.31, p=.042, \eta_p^2=.07$), but did not interact with Generation ($F(1, 62)=0.02, p=.893, \eta_p^2<.001$). Unrelated to nocebo hyperalgesia, there was a main effect of Trial ($F(2, 124)=15.67, p<.001, \eta_p^2=.20$), with anticipatory EDA habituating over time. No other statistically significant effects were observed (all *ps*>.05).

Anticipatory Outcomes: Expectancy Ratings

Participants expected more pain when presented with the Tx (M_{adj}=45.84, *SEM*=2.34) relative to NT (M_{adj}=34.89, *SEM*=2.27) Cue ($F(1, 62)=19.96, p<.001, \eta_p^2=.24$), see Figure 2. There were no other main effects or interactions (all *ps*>.05), including between Generation and Cue ($F(1, 62)=1.35, p=.249, \eta_p^2=.02$), suggesting expectancies were reliably passed along the transmission chain.

Heat-Evoked Responses: FAU Heat

As depicted in Figure 2, pain-related FAUs were found to be elevated on Tx (M_{adj}=1.72, *SEM*=0.17) relative to NT (M_{adj}=1.40, *SEM*=0.12) Trials, despite equivalence in the intensity of the nociceptive stimuli delivered ($F(1, 62)=7.28, p=.009, \eta_p^2=.11$). There was a Generation by Trial interaction ($F(2,124)=3.07, p=.050, \eta_p^2=.05$) that reached threshold statistical significance, with the effect of Trial only differing for G2 (p=.001) and not G3 (p=.649), and only at T2 vs. T3 (p<.001) where FAUs decreased at the end of the Block. There was also a Gender by Trial interaction ($F(2,124)=3.86, p=.024, \eta_p^2=.06$). The effect of Trial was marginally significant for gender-matched dyads (p=.050) but not for unmatched dyads (p=.107), with none of the individual trials significantly differing when decomposed (all *ps*>.05). However, neither of these interactions concerned nocebo

hyperalgesia. No other statistically significant main effects or interactions were observed, including Generation by Cue ($F(1, 62)=0.03$, $p=.852$, $\eta_p^2<.001$).

Heat-Evoked Responses: EDA Heat

Other than a main effect of Trial ($F(2, 124)=7.13$, $p=.001$, $h_p^2=.10$), where EDA habituated over time, there were no significant main effects and interactions (all *ps*>.05).

Supplementary Table 1:
Hierarchical Regression Models including the pain difference of the Demonstrator (Treatment minus No Treatment Pain Ratings) and Psychological Synchrony (Psych Synchrony) between Demonstrator and Observer dyads on the magnitude of Nocebo Hyperalgesia (Treatment minus No Treatment Pain Ratings) when the Observer in the dyad subsequently became the Observer. Model 1 includes the pain difference for the Generation 1 (G1) Demonstrator, and Psychological Synchrony in Expectancy Ratings between Generation 1 and Generation 2 dyads. Model 2 presents the same statistics, but for the Generation 2 (G2) Demonstrator's pain difference, and Psychological Synchrony between Generation 2/Generation 3 dyads. Models were run on data where full transmission existed (G1-G2-G3) and therefore both models have N=28 dyads (two participants removed due to missing synchrony data)

Model 1: G1(Demonstrator)/G2(Observer) Dyads (Outcome=G2 nocebo hyperalgesia (Tx-NT))

	Model Summary / ANOVA				Coefficients				
	R^2	$R^2_{adjusted}$	F	p	B	SE	β	t	p
**Step 1**	.03	-.01	0.81	.379					
Pain Difference (G1)					0.14	0.15	0.18	0.91	.370
**Step 2**	.04	-.04	0.24	.631					
Pain Difference (G1)					0.15	0.16	0.19	0.95	.350
Psych Synchrony (G1/G2)					-1.37	2.81	-0.10	0.49	.630
**Step 3**	.08	-.04	0.99	.353					
Pain Difference (G1)					0.23	0.18	0.30	1.30	.208
Psych Synchrony (G1/G2)					-9.07	8.59	-0.65	1.06	.302
PainDiff:Psych Synchrony					0.18	0.19	0.58	0.95	.353

Model 2: G2(Demonstrator)/G3(Observer) Dyads (Outcome=G3 nocebo hyperalgesia (Tx-NT))

	Model Summary / ANOVA				Coefficients				
	R^2	$R^2_{adjusted}$	F	p	B	SE	β	t	p
**Step 1**	.24	.21	6.41	**.001**					
Pain Difference (G2)					0.47	0.17	0.49	2.80	**.010**
**Step 2**	.47	.43	15.92	**.001**					
Pain Difference (G2)					0.31	0.15	0.32	2.06	**.050**
Psych Synchrony (G2/G3)					0.98	2.16	0.51	3.24	**.004**
**Step 3**	.67	.62	3.49	**.001**					
Pain difference (G2)					0.29	0.12	0.31	2.42	**.024**
Pain Prediction (G2/G3)					0.72	2.44	0.05	0.29	.772
PainDiff:Psych Synchrony					0.47	0.13	0.64	3.67	**.001**

Supplementary Note 3: Responder Status on Secondary Outcomes

To test whether Responders and Non-Responders existed in the dataset, Monte Carlo permutation analysis was performed on participants pain ratings. All G1-Demonstrators were classified as Responders. A total of 16 G2-Demonstrators and 13 G3-Demonstrators were classified as Responders (20/16 Non-Responders, respectively). The proportion of Responders/Non-Responders did not differ significantly across G2 and G3 groups ($\chi^2(1,N=65)=0.001$, $p=.975$, Cramer's $V=.004$) suggesting that there was limited significant change in receptivity to the manipulation at later stages of the chain. Of the Non-Responders, a minority ($N_{G2}=2$; $N_{G3}=6$) were able to be classified as 'Reverse-Responders': demonstrating elevated Pain-Ratings to NT. All of these Reverse-Responders identified the incorrect colour as the Tx Cue, suggesting a misinterpretation of the social information provided by the demonstrator. Reverse-Responders were not significantly more likely in either Generation ($\chi^2(2,N=65)=3.89$, $p=.138$, Cramer's $V=.25$). There was an effect of Responder on TENS Confidence ($F(1, 62)=4.09$, $p=.022$, $\eta_p^2=.12$). Responders had higher confidence ($M=5.13$, $SE=0.31$, 95%CI [4.53, 5.75]) than Non-Responders ($M=3.89$, $SE=0.31$, 95%CI [3.27, 4.52]) when identifying the TENS Cue ($p=.016$). This result seems intuitive as Non-Responders reported experiencing a limited difference in pain between conditions. Reverse-Responders ($M=4.63$, $SE=0.58$, 95%CI [3.46, 5.79]) did not differ from Responders or Non-Responders (both $ps>.50$).

Interactions between Responder-Status and Cue were found for Expectancy ($F(1, 62)=49.40$, $p<.001$, $\eta_p^2=.44$; Responder (Tx>NT; $p<.001$); Non-Responder (Tx≠NT; $p=.864$)) and FAU_{heat} ($F(1, 62)=14.93$, $p<.001$, $\eta_p^2=.19$; Responder (Tx>NT; $p<.001$); Non-Responder (Tx≠NT; $p=.737$)). A similar pattern was found for EDA_{heat} ($F(1, 62)=15.52$, $p<.001$, $\eta_p^2=.20$). However, both Responders (Tx>NT; $p=.002$) and Non-Responders (Tx<NT; $p=.029$) differed by Cue. The effect for Non-Responders was driven by the presence of 'Reverse-Responders' and was no longer significant with these participants removed ($p=.405$). The interaction neared significance for EDA_{cue} ($F(1, 62)=3.29$, $p=.074$, $\eta_p^2=.05$; Responder (Tx>NT; $p=.007$); Non-Responder (Tx≠NT; $p=.712$)). For FAU_{cue} there was an interaction between Responder, Cue, and Trial ($F(1, 124)=3.54$, $p=.032$, $\eta_p^2=.05$). However, the only Tx vs NT difference was at T3 where Non-Responders had higher anticipatory FAUs for NT Cues ($p=.017$). Overall, Responders expressed pain-related information while Non-Responders did not. Supplementary Figure 4 presents the mean scores for those classified as Responders and Non-Responders.

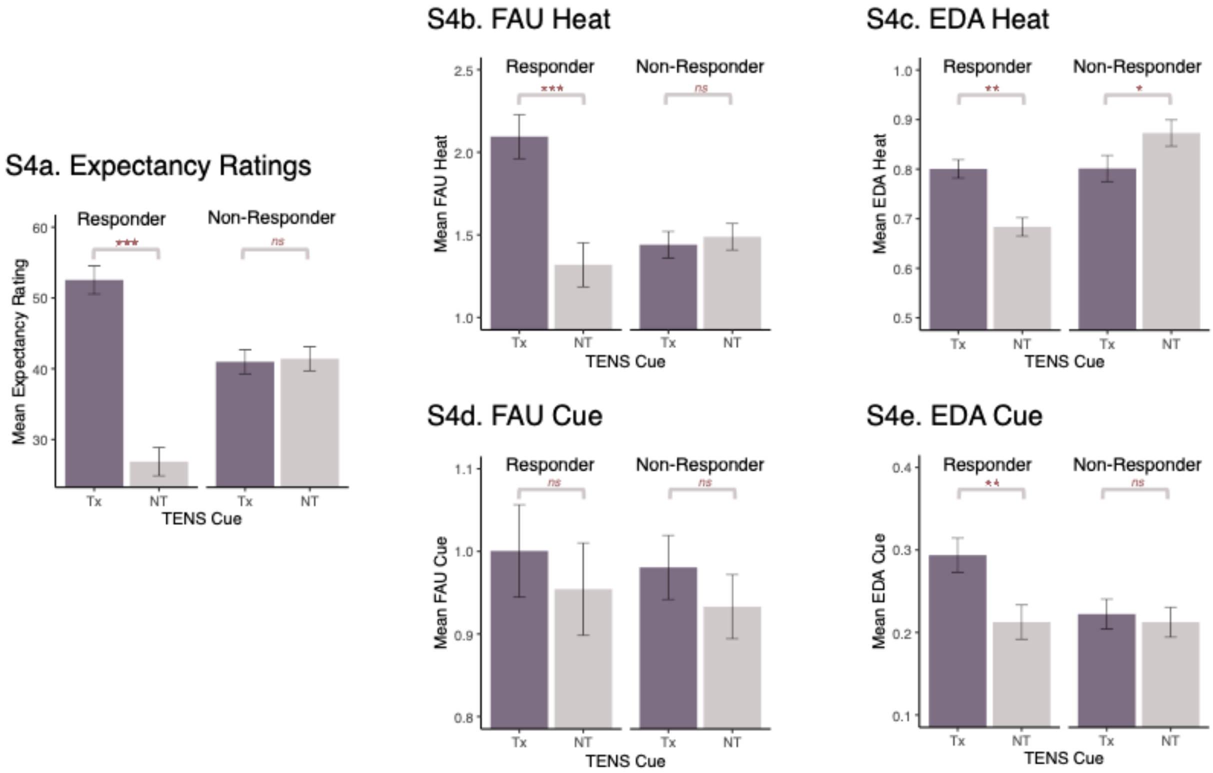

Supplementary Figure 4:
Mean Responder vs. Non-Responder responses (collapsed across G2 and G3 participants; *N*=65) on Tx (‘Treatment’) and NT (‘No Treatment’) trials (both paired with the Low Temperature Destination; 45°C). Error bars represent +/- 1SED, adjusted for the within-subjects comparison (using the afex package in R). Significance levels are depicted as *** ($p<.001$); ** ($p<.01$), and * ($p<.05$)

Supplementary Note 4: Observer Responses to Demonstrators

Observer Expectancy Ratings

As depicted in Article Figure 5a., there was a Generation by Cue interaction ($F(1, 58)=5.98$, $p=.018$, $\eta_p^2=.09$) on Expectancy Ratings. However, the difference between Tx and NT was significant for both Generations (G2: $p<.0001$ | G2: $p=.019$). There was also a Cue by Trial interaction ($F(2, 116)=11.42$, $p<.001$, $\eta_p^2=.16$), where Expectancy ratings for Tx were significantly lower for T1 relative to T2 ($p=.003$) and T3 ($p=.031$) Trials.

FAU Cue and EDA Cue

As depicted and Figure 5d. and 5e., there was no effect of Cue on FAU_{cue} or EDA_{cue}, nor were there any interactions (all *ps*>.05).

FAU Heat

As depicted in Figure 5b., there was a Generation by Cue interaction on Observers FAUs when the thermal stimulus was delivered to the Demonstrator ($F(1, 62)=4.45$, $p=.039$, $\eta_p^2=.04$), where Tx differed from NT for G2 ($p<.001$) but not G3 ($p=.080$) participants. There was also a main effect of Trial ($F(2, 124)=9.82$, $p<.001$, $\eta_p^2=.14$) where FAUs habituated over time.

EDA Heat

As depicted in Figure 5c., there was a Generation by Cue interaction on Observers' EDA response to delivery of the thermal stimulus ($F(1, 62)=10.62$, $p=.002$, $\eta_p^2=.15$). Tx differed from NT for G2 ($p<.001$) but not G3 ($p=.220$) participants. There was also a main effect of Trial ($F(2, 124)=20.29$, $p<.001$, $\eta_p^2=.25$) where EDA habituated over Trials.

Generation 3 Observers

The magnitude of responses differed between G2 and G3 as Observers, with Expectancy reduced, and EDA_{heat} and FAU_{heat} non-significant, in the latter Generation. However, all G2-Observers watched a G1-Demonstrator who was a 'Responder', whereas the same applied to less than half of G3-Observers (those watching a Responder $N=14$; Non-Responder $N=15$). A further analysis was therefore run on G3-Observers alone. Whether the G2-Demonstrator in their dyad was a 'Responder' vs. 'Non-Responder' was added to the model. For Expectancy, there was a significant 'Responder' by Cue interaction ($F(1, 25)=12.24$, $p=.002$, $\eta_p^2=.33$). Expectancies differed by Cue when observing a 'Responder' ($p<.001$), but not 'Non-Responder' ($p=.669$). For EDA_{heat} there was a three-way interaction between 'Responder', Cue, and Trial ($F(2, 52)=4.50$, $p=.016$, $\eta_p^2=.15$). The difference between Tx and NT Cues was significant across all three trials when watching a 'Responder' (all *ps*<.05), with differences becoming numerically larger over successive trials. This was not the case when watching a 'Non-Responder' (all *ps*>.05). Finally, there was no 'Responder'

by Cue interaction for FAU_{heat}, but when G3-Observers were analysed alone, there was a significant effect of Cue ($F(1, 26)=4.92$, $p=.036$, $\eta_p^2=.16$), with larger responses to Tx. As such, the lack of expression of empathetic pain among those in G3 may be largely explained by whether they were observing a Demonstrator who expressed increased nocebo hyperalgesia or not.

Supplementary Table 2:

Hierarchical regression output for the effect of Generation 2 and Generation 3 Observer's (N=65) empathetic responses (difference scores, Treatment minus No Treatment, for: Expectancy, FAU_{heat}, and EDA_{heat}) on the magnitude of their subsequent nocebo hyperalgesic response (difference score: Pain Ratings, Treatment minus No Treatment) when Demonstrating.

Observer Expectancy Ratings on Nocebo Hyperalgesia

	Model Summary / ANOVA				Coefficients				
	R^2	$R^2_{adjusted}$	F	p	B	SE	β	t	p
**Step 1**	.16	.15	12.62	**<.001**					
Observer Response					0.24	0.07	0.41	3.49	**<.001**
**Step 2**	.17	.14	0.10	.749					
Observer Response					0.23	0.07	0.40	3.20	**.002**
Generation					0.52	1.68	0.04	0.31	.755
**Step 3**	.22	.18	4.26	**.043**					
Observer Response					0.23	0.07	0.39	3.25	.002
Generation					3.14	2.07	0.23	1.52	.134
Observer:Generation					-0.15	0.07	-0.30	2.06	**.044**

Observer FAU Heat on Nocebo Hyperalgesia

	Model Summary / ANOVA				Coefficients				
	R^2	$R^2_{adjusted}$	F	p	B	SE	β	t	p
**Step 1**	.08	.07	5.28	**.025**					
Observer Response					4.77	2.06	0.28	2.32	**.024**
**Step 2**	.09	.06	0.56	.459					
Observer Response					4.37	2.05	0.26	2.05	**.045**
Generation					1.29	1.72	0.10	0.75	.456
**Step 3**	.09	.05	0.24	.628					
Observer Response					4.84	2.35	0.29	2.06	.044
Generation					1.75	1.97	0.13	0.89	.379
Observer:Generation					-1.14	2.35	-0.08	0.49	.628

Observer EDA Heat on Nocebo Hyperalgesia

	Model Summary / ANOVA				Coefficients				
	R^2	$R^2_{adjusted}$	F	p	B	SE	β	t	p
**Step 1**	.01	-.01	0.39	.536					
Observer Response					-3.53	6.03	-0.07	0.59	.560
**Step 2**	.05	.02	2.99	.089					
Observer Response					-7.77	6.48	-0.16	1.20	.235
Generation					3.06	1.86	0.23	1.65	.105
**Step 3**	.15	.11	7.01	**.010**					
Observer Response					2.63	7.32	0.06	0.36	.721
Generation					5.16	1.94	0.38	2.66	.010
Observer:Generation					-19.38	7.32	-0.45	2.65	**.010**
