# Supplementary material for: Interpersonal physiological and psychological synchrony predict the social transmission of nocebo hyperalgesia between individuals
Source: Commun Psychol. 2024 Apr 29;2:33. doi: 10.1038/s44271-024-00069-6 (PMC11332037; doi:10.1038/s44271-024-00069-6)
Supplement: Supplementary file 3 — Reporting Summary [file 44271_2024_69_MOESM3_ESM.pdf]

nature portfolio

Corresponding author(s): Kirsten Barnes

Last updated by author(s): 10/12/2023

Reporting Summary

Nature Portfolio wishes to improve the reproducibility of the work that we publish. This form provides structure for consistency and transparency in reporting. For further information on Nature Portfolio policies, see our Editorial Policies and the Editorial Policy Checklist.

Statistics

For all statistical analyses, confirm that the following items are present in the figure legend, table legend, main text, or Methods section.

n/a	Confirmed	
☐	☒	The exact sample size (*n*) for each experimental group/condition, given as a discrete number and unit of measurement
☐	☒	A statement on whether measurements were taken from distinct samples or whether the same sample was measured repeatedly
☐	☒	The statistical test(s) used AND whether they are one- or two-sided<br>*Only common tests should be described solely by name; describe more complex techniques in the Methods section.*
☐	☒	A description of all covariates tested
☐	☒	A description of any assumptions or corrections, such as tests of normality and adjustment for multiple comparisons
☐	☒	A full description of the statistical parameters including central tendency (e.g. means) or other basic estimates (e.g. regression coefficient) AND variation (e.g. standard deviation) or associated estimates of uncertainty (e.g. confidence intervals)
☐	☒	For null hypothesis testing, the test statistic (e.g. *F*, *t*, *r*) with confidence intervals, effect sizes, degrees of freedom and *P* value noted<br>*Give P values as exact values whenever suitable.*
☒	☐	For Bayesian analysis, information on the choice of priors and Markov chain Monte Carlo settings
☐	☒	For hierarchical and complex designs, identification of the appropriate level for tests and full reporting of outcomes
☐	☒	Estimates of effect sizes (e.g. Cohen's *d*, Pearson's *r*), indicating how they were calculated

Our web collection on statistics for biologists contains articles on many of the points above.

Software and code

Policy information about availability of computer code

Data collection	Psychopy3<br>LabChart v8
Data analysis	R (4.3.1) / R Studio (2023.06.1)<br>MATLAB v2022a<br>OpenFace 2.2.0

For manuscripts utilizing custom algorithms or software that are central to the research but not yet described in published literature, software must be made available to editors and reviewers. We strongly encourage code deposition in a community repository (e.g. GitHub). See the Nature Portfolio guidelines for submitting code & software for further information.

Data

Policy information about availability of data

All manuscripts must include a data availability statement. This statement should provide the following information, where applicable:
- Accession codes, unique identifiers, or web links for publicly available datasets
- A description of any restrictions on data availability
- For clinical datasets or third party data, please ensure that the statement adheres to our policy

Data and code is available via the Open Science Framework repository: https://osf.io/qzstu/?view_only=83bba82b3c3c44818dede45feb1c53cc

Human research participants

Policy information about studies involving human research participants and Sex and Gender in Research.

Reporting on sex and gender	Participants self-reported their gender only. Self-report options were: female, male, non-binary/third gender, prefer not to say, other (free-response)). However, all participants identified with the first three options. The gender of the two participants in each dyad was used as a covariate in the primary analyses, given a previously reported association between gender and socially acquired nocebo effects. This was coded as gender match vs. gender mismatch calculated from the participants self-report.
Population characteristics	See below
Recruitment	Participants were either University of Sydney students (recruited via SONA in return for partial course credit) or were recruited through Facebook (in return for $30 AUD). In both cases, participants saw an advert containing a short generic description of the study. In neither case were participants directly approached. Given that pain was mentioned as part of this advert, it is possible that participants were self-selecting, given that those unwilling to experience pain would not have responded. The consequence being, that those who find pain highly aversive may not have been included in the sample. From an ethical standpoint, however, omission of this information would not have been possible and the same participants would likely have withdrawn.
Ethics oversight	University of Sydney (Human Research Ethics Committee).

Note that full information on the approval of the study protocol must also be provided in the manuscript.

Field-specific reporting

Please select the one below that is the best fit for your research. If you are not sure, read the appropriate sections before making your selection.

☐ Life sciences ☒ Behavioural & social sciences ☐ Ecological, evolutionary & environmental sciences

For a reference copy of the document with all sections, see nature.com/documents/nr-reporting-summary-flat.pdf

Behavioural & social sciences study design

All studies must disclose on these points even when the disclosure is negative.

Study description	We report on one experiment where behavioural ratings regarding subjective pain (VAS measurement), autonomic arousal (electrodermal activity), and facial action units (collected via webcam recordings) were collected. Data are quantitative.
Research sample	One hundred and one healthy volunteers, eighteen years of age or over (70 identified as female, 24 as male, and 7 as non-binary; mean age=23.7 years). Pain-related exclusion criteria were employed to screen participants (see manuscript).
Sampling strategy	Convenience/availability sampling (participants signed up to participate, and were tested if they fulfilled the inclusion criteria).
Data collection	Behavioral data (pain ratings) were collected via a visual analogue scale. EDA data were digitized using a PowerLab 2/26 DAQ device. Facial action units were collected via a 1920 × 1080p, 60fps webcam. Testing occurred in an enclosed room with only the two participants in each dyad and the experimenter present. The other participants waited in a dedicated waiting room with room dividers to prohibit conferral. The experimenter was not blind to the study hypotheses during data collection, but they were kept blind to the colour of the cue that denoted sham TENS 'activity', meaning that they could not transmit this information to the participant. Once the experimental equipment was set up, participants were left to complete the task without interruption. Data extraction and analysis was performed by researchers not involved in the data collection process.
Timing	29th May - 23rd August 2022
Data exclusions	No data exclusions
Non-participation	NA
Randomization	Participants were randomised to the one of the three conditions (Generations 1-3) via a random number generator.

Reporting for specific materials, systems and methods

We require information from authors about some types of materials, experimental systems and methods used in many studies. Here, indicate whether each material, system or method listed is relevant to your study. If you are not sure if a list item applies to your research, read the appropriate section before selecting a response.

Materials & experimental systems

n/a	Involved in the study
☒	☐ Antibodies
☒	☐ Eukaryotic cell lines
☒	☐ Palaeontology and archaeology
☒	☐ Animals and other organisms
☒	☐ Clinical data
☒	☐ Dual use research of concern

Methods

n/a	Involved in the study
☒	☐ ChIP-seq
☒	☐ Flow cytometry
☒	☐ MRI-based neuroimaging
